# Modern Medical Therapeutics and Modern Surgical Therapeutics

**Published:** 1877-12

**Authors:** 


"MODERN MEDICAL THERAPEUTICS" and "MODERN SURGICAL THERAPEUTICS." By George H. Napheys, A.M., M.D., etc. From the publishing house of D. G. Brinton, Philadelphia, 1877. For each work, price, post-paid, in Cloth, $4.00; Leather, $5.00.

In these two volumes of 580 pages each, pathology, etiology and diagnosis, as well as the action of remedies, are absolutely ignored, and diseases by name are associated with the name of remedies singly, and in the form of formula for combinations. The two volumes are, therefore, formularies of medicine and surgery.

A vaulable library might be made of Zeimssen's Practice of Medicine, Napheys' Modern Medical and Surgical Therapeutics, with the addition of some reliable work on the action of remedies, but the empirical and routine manner of prescribing for disease, to which formularies inevitably lead the practioner, is the most serious hindrance to the progress of rational medicine. Students, beginners and old practitioners, eager to arrive at the ultimate object of all medical investigations, are too often inclined to sooth their consciences into the acceptance of the indolent habit of emperical prescriptions.

Cod liver oil for phthisis, quinine for malarial fever, mercury for dysentery, etc., etc., may be learned by rote, as the school boy learns his selection for declamation, or with formulary under arm, as did the Thompsonian steam doctor, in the days of my boyhood, they may go forth, but complications, varied stages and errors of diagnosis, render such practitioners not only useless, but dangerous to human life.

